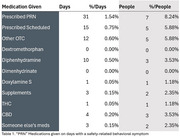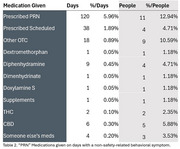# Medication Administration Practices and Chemical Restraint Use by Dementia Family Caregivers

**DOI:** 10.1002/alz70861_108843

**Published:** 2025-12-23

**Authors:** Carolyn Pickering, Vicki Winstead, Wesley R Browning

**Affiliations:** ^1^ University of Texas Health Science Center at Houston, Houston, TX USA; ^2^ UTHealth Houston, Houston, TX USA

## Abstract

**Background:**

The presentation explores the practices of medication administration and the potential use of chemical restraints by family caregivers of individuals with dementia. Chemical restraint is defined as any drug used for discipline or convenience rather than to treat a person’s medical symptoms. The prevalence of chemical restraints in nursing homes has decreased due to CMS programs, but caregivers play a critical role in managing medications for persons with Alzheimer's disease and related dementias (ADRD) .

**Methods:**

This paper reviews preliminary data from 85 family caregiving dyads and over 2,012 daily observations. Complete medication lists were gathered at enrollment, and caregivers completed 31 days of daily surveys reporting on behavioral symptoms and the pharmacological and non‐pharmacological strategies used to manage these symptoms. The frequency of PRN medication usage, types of medications administered and potential chemical restraint were determined.

**Results:**

The findings indicate that 42% of caregivers administered a “PRN” medication over 31 days, with PRN medications given on 18% of days. Safety‐related behavioral symptoms were reported on 9% of days, and PRN medications were given on 45% of those days. Non‐safety‐related behavioral symptoms were reported on 70% of days, with PRN medications given on 14% of those days. Importantly, about half of medications given as “PRN” by caregivers were not prescribed as such but included additional dosages of scheduled medications, over‐the‐counters (i.e., Benadryl), CBD/THC and drug divergence. We estimate approximately 15% of caregivers were using chemical restraints, and 16% were using potentially inappropriate medication practices.

**Conclusions:**

These findings underscore the urgent need to address informal chemical restraint practices occurring in home settings, where oversight is limited and caregivers may lack adequate guidance. The use of non‐prescribed substances and potentially inappropriate medications to manage behavioral symptoms highlights significant gaps in caregiver support and education. Interventions that promote safe medication management such as deprescribing initiatives, tailored caregiver training, and programs to build non‐pharmacologic coping strategies are critically needed. Future work should prioritize the development of tools to identify chemical restraint in community settings and evaluate scalable strategies that empower caregivers while protecting the dignity and well‐being of persons living with dementia.